# A prospective cohort of treatment-seeking patients with problematic use of prescription narcotic drugs: study protocol and baseline characteristics

**DOI:** 10.1186/s12888-024-06368-w

**Published:** 2024-12-20

**Authors:** Sofia Burmester, Cecilia Krüger, Jonas Hällgren, Jeanette Westman, Johan Franck

**Affiliations:** 1 Department of Neurobiology, Care Sciences and Society, Karolinska Institutet, Alfred Nobels allé 23, Huddinge, 141 52 Sweden; 2 The Stockholm Centre for Dependency Disorders, Stockholm Health Care Services, Region Stockholm, Friskvårdsvägen 4, Stockholm, 112 81 Sweden; 3 Academic Primary Care Centre, Region Stockholm, Solnavägen 1 E, Stockholm, 113 65 Sweden; 4 Department of Health Care Sciences, Marie Cederschiöld University, Folkungagatan 127, Stockholm, 116 30 Sweden; 5 Department of Clinical Neuroscience (CPF), Karolinska Institutet, Norra stationsgatan 69, Stockholm, 113 64 Sweden

**Keywords:** Analgesics, opioid, Benzodiazepines, Prescription drugs, Substance-related disorders, Prescription drug misuse, Treatment outcome

## Abstract

**Background:**

There is limited knowledge on long-term outcomes of tapering treatment for individuals with problematic use of prescription narcotics, including opioids and benzodiazepines. The overall aim of the study is to investigate clinical trajectories and treatment outcomes of patients seeking treatment in addiction care.

**Methods:**

This paper presents the study protocol and baseline characteristics of a cohort of patients seeking treatment for problematic use of prescription narcotic drugs at specialized outpatient addiction services. Treatment for addiction at the clinic includes drug tapering and treatment for underlying psychiatric disorders. Data in this prospective cohort study is collected from biomarkers, self-report questionnaires, patient medical records, and national registers at baseline and follow-up visits at 6, 12, and 24 months.

**Results:**

A clinical cohort of 405 participants were enrolled in the study between 2018 and 2023. The study population (57.5% women; 42.5% men) at baseline had a mean age of 49.2 years (SD = 14.0). Participants in the cohort had used prescription narcotics for 11 years on average before seeking treatment, with opioid analgesics (66.2%) being the most common at baseline, followed by benzodiazepines (50.9%). Most participants (75.9%) had received prescription narcotics from their health care provider, although illegal sources were common. Besides substance use disorders, many also had anxiety disorders (46.3%) and depression (40.4%) at baseline. Previous treatment for problematic alcohol or drug use were reported by 14.0% and 21.6%, respectively.

**Discussion:**

This prospective, naturalistic cohort will provide novel information on long-term outcomes of tapering treatment and identify prognostic factors for treatment success, including abstinence. Future papers will investigate individual and treatment-related characteristics of the patient population. Baseline data suggest that many patients with problematic use of prescription narcotics receive prescriptions over many years from their regular health care providers, which contradicts most clinical guidelines.

**Trial registration:**

NCT03713983 22/10/2018.

## Background

Prescription drugs such as opioid analgesics and benzodiazepines (including benzodiazepine-related z-drugs) are used in the treatment of pain, anxiety, and insomnia. Such drugs, classified as narcotics in Sweden, have been associated with adverse outcomes including problematic use (defined as non-medical use, misuse, addiction, dependence, or substance use disorders) [1, 2]. In Sweden, over 1 million prescriptions of narcotic drugs were issued in 2023 [3], and the prevalence of problematic use has been estimated to 1 to 2% of the population [4, 5]. The risk of adverse outcomes associated with prescription narcotic drugs has led to restrictive clinical guidelines and prioritization of other treatments [2, 6–10]. However, opioid analgesics and benzodiazepines are still commonly prescribed and used long-term [11, 12], placing individuals at risk of adverse outcomes. Tapering by gradual dose reduction and treatment for underlying psychiatric disorders are central treatment components for individuals with problematic use of opioid analgesic medications and benzodiazepines [2, 13, 14]. Maintenance treatment is another evidence-based treatment for opioid dependence [2, 13, 14]. In Sweden, tapering and maintenance treatment have the same priority of evidence for the treatment of opioid analgesic dependence [13].

The clinical trajectories of patients during and following tapering treatment in addiction care are not well documented as there are few previous studies on individuals with problematic use. In addition, most studies evaluating outpatient tapering in individuals with problematic use of opioid analgesics or benzodiazepines typically have fewer than 100 participants. To the authors’ knowledge, only one tapering study on benzodiazepines [15] and one on opioids [16] have included more than 200 individuals. There are also variations in the treatment evaluated, with tapering regimens spanning from weeks to months [16–34], or individual, symptom-guided tapering lasting for over a year [31, 35]. While some studies have solely evaluated tapering treatment, others have evaluated tapering with adjunct pharmacological treatment [15, 22–26, 36] or psychotherapy [16, 17, 28–31], both of which seem to provide minimal additional benefit to tapering outcomes.

The outcome of tapering treatment is often evaluated by estimating the proportion of patients who are abstinent from prescription drug use at follow-up. Time to follow-up in previous studies varies between the end of treatment and up to 5 years, with follow-up of 6 months or less being most reported. Abstinence has been measured in different ways, with toxicology tests [15–21, 25, 26, 30–32, 37] and self-report [22, 33, 38–41] being the most common. In some studies, the method of abstinence evaluation is unspecified [23, 24, 27–29, 35]. There is a wide range in the reported proportion of abstinence in previous studies, ranging from 6 to 85%, with a majority reporting less than 50% abstinence by end of study.

Previous studies on tapering also demonstrate variability in the factors associated with patient outcomes, showing either non-significant or mixed results. Common examples of investigated factors include baseline dose [23, 26, 30, 35, 39, 41, 42], duration of use [30, 35, 39], treatment duration [35, 37], sex [27, 35, 39, 43], age [35, 39], psychiatric symptoms [25, 27, 30, 42], and previous tapering attempts [39, 41, 44].

The lack of large studies and the mixed results from previous research calls for studies with larger cohorts, longer follow-up time, and objective outcome measures. The variability in previous study methodologies, including the measurement of abstinence, type of treatment (tapering with or without add-on treatment), duration, and the lack of intention-to-treat design precludes conclusions regarding treatment outcomes and best practices. More knowledge is needed about long-term outcomes and prognostic factors which can form the basis for improved treatment and policies for individuals with problematic use of prescription narcotics.

### Aim of the study

The overall aim of the study was to investigate clinical trajectories and treatment outcomes of patients seeking treatment for problematic use of prescription narcotics. The study focuses on opioid analgesics and benzodiazepines. This paper describes the study protocol and baseline characteristics of the study population.

## Methods

### Study design

This is an ongoing prospective, naturalistic cohort study of patients seeking treatment for problematic use of prescription narcotic drugs at a specialized outpatient addiction clinic. In this study, problematic use was assessed by a clinician according to two sets of diagnostic criteria: harmful use or dependence according to International Statistical Classification of Diseases and Related Health Problems 10th Revision (ICD-10) and substance use disorder according to the Diagnostic and Statistical Manual of Mental Disorders, fifth edition (DSM-5). Study materials include self-report questionnaires, diagnostic interviews, patients’ medical records, and biomarkers. Drug abstinence will be assessed from data on prescription narcotic drug use and toxicology test results. The clinical follow-up period is 24 months. In addition, a 10-year follow-up is planned using national registers administered by the Swedish National Board of Health and Welfare.

The study was approved by the Regional Ethics Board in Stockholm (Dnr. 2018/985 − 31/1) and was registered on ClinicalTrials.gov (NCT03713983). The study adheres to the principles for the protection of the rights of study participants outlined in the Declaration of Helsinki and includes third-party monitoring by Karolinska Trial Alliance to ensure adherence to the study protocol and good data quality.

### Setting

The study is conducted at an urban outpatient addiction clinic in Sweden that specializes in treatment of problematic use of prescription narcotic drugs. Patients include those with a prescription as well as those who obtain narcotics by non-prescription means (e.g., from friends, relatives, or purchased via the internet or local illegal market). Patients seek treatment at the clinic by self-referral or referral from other health care services. Prior to initiating tapering treatment patients receive a clinical evaluation of their somatic and psychiatric health, including substance use. Participants in this study receive standard care based on national and regional guidelines, which consists of tapering with the addition of psychological and/or pharmacological treatment of comorbid psychiatric disorders if needed. The tapering protocol is patient-centered, including joint patient-provider decision making and close monitoring through in-person visits with health care personnel. Patients often taper the substance that they have used; however, some patients may be switched to different substances in the same class or extended-release formulations to help manage symptoms. Individuals with poly-substance use often begin tapering on opioids. In the tapering schedule, doses are split evenly over the course of the day to minimize withdrawal symptoms. In the case of worsening symptoms or substance use, tapering can be paused at the same dose level to allow for stabilization before tapering continues. The study did not test any new treatments, nor did it evaluate maintenance treatment for individuals with opioid dependence.

### Participants

**Fig. 1:**
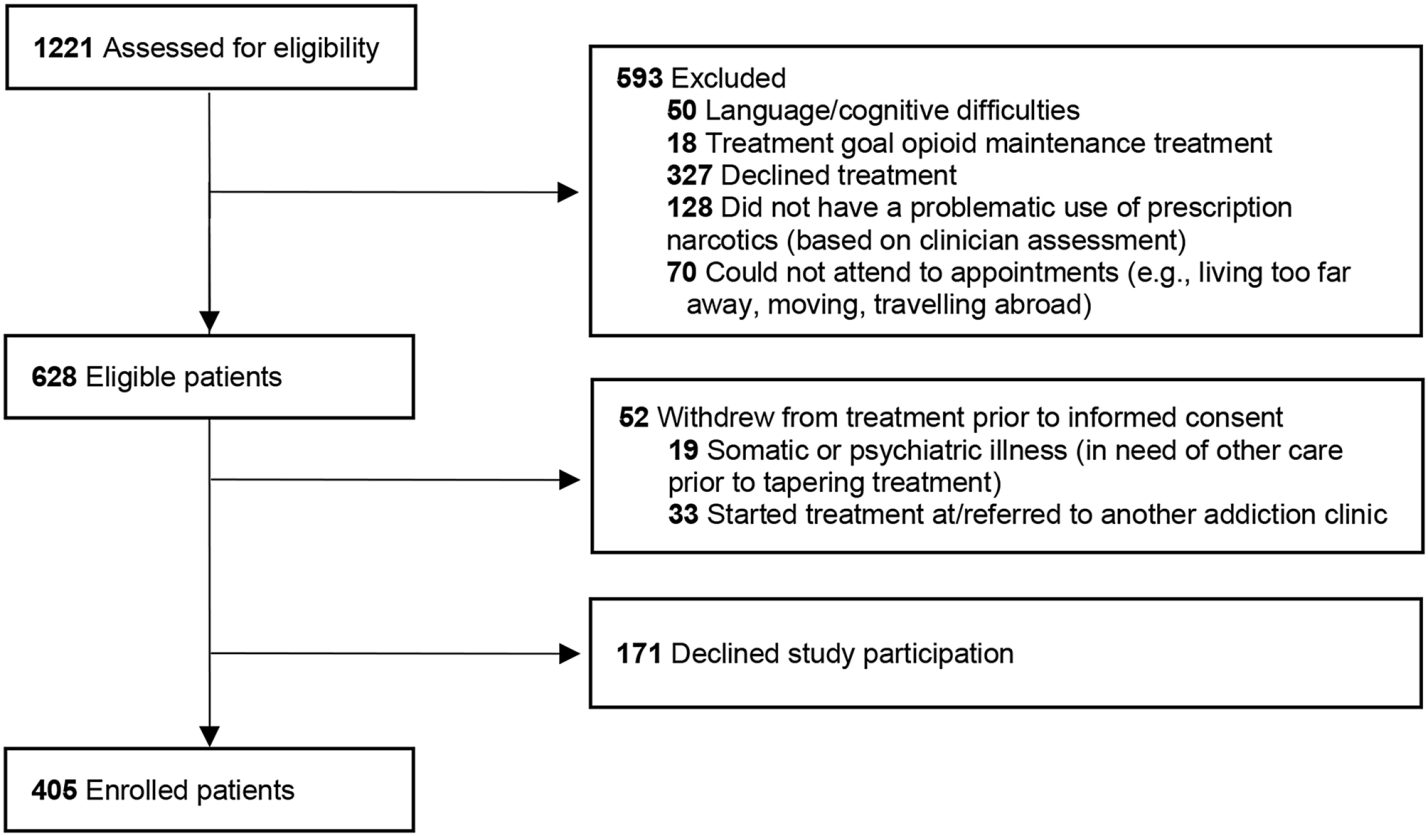
Flowchart of patients visiting the clinic during the study period, screening criteria, and study enrollment

During the enrollment period, October 2018 to April 2023, a total of 1221 patients with at least one visit at the outpatient clinic received verbal and written information about the study and were assessed for eligibility. Individuals who were 18 years or older, were seeking treatment for problematic use of prescription narcotic drugs, and who had not started tapering treatment at the clinic prior to study screening were eligible to participate. Non-Swedish speaking patients and individuals with cognitive difficulties (i.e., unable to give informed consent), and whose treatment goal was maintenance treatment, were excluded. Moreover, individuals who did not progress to treatment (e.g., declined treatment or could not attend to appointments) were not eligible for the study (see Fig. 1). Eligible patients had the opportunity to discuss participation with the recruiting health care provider before providing written informed consent. Participation in the study did not influence the treatment offered or provided at the clinic. A total of 628 patients met eligibility criteria, and 405 were enrolled in the study, resulting in an inclusion rate of 64.5% of eligible patients.

### Outcomes

The primary outcome is change in prescription narcotic drug use, defined as discontinued prescription at 12 months. Secondary outcomes are: absence of drug on supervised toxicology testing at the 12-month follow-up and retention in treatment, defined as the number of participants who have completed tapering or remain in treatment at follow-ups. Other outcome measures will also be investigated.

### Procedure

Data is collected at baseline and follow-up visits, conducted at 6, 12, and 24 months after enrollment. All participants are invited to follow-up visits, including individuals whose clinical contact is completed before the end of the study. Patients are compensated with a 450 SEK grocery-store gift card at each follow-up visit.

### Instruments

Study data were collected at baseline and will be collected at follow-up visits from study assessments, medical records, and the National Prescribed Drug Register, which is a nationwide population register encompassing complete information on all pharmacy-dispensed prescriptions in Sweden [45] (see Table 1). Self-report data include assessments on sociodemographic background and medical history. Psychiatric symptoms are assessed by self-rated and clinician-rated instruments. Substance use is inferred from several sources, including in blood, urine, and saliva biomarkers as well as from medical records and the National Prescribed Drug Register.

**Table 1:** Study assessments at baseline and follow-ups, including instrument or data source, rater, and time point

Assessment	Instrument/source	Rater	Time point
Patient characteristics (e.g., age, sex, living situation, education, occupation, children, physical activity)	Questionnaire	Self-rated	Baseline
Prescription narcotic drug use (e.g., type of drug, duration of use, provider)	Questionnaire	Self-rated	Baseline
Previous treatment for problematic alcohol and drug use	Questionnaire	Self-rated	Baseline
Presence of psychiatric diagnoses	Questionnaire Mini International Neuropsychiatric Interview, Version 7 (M.I.N.I.) [46, 47]	Self-rated Clinician-rated	Baseline
Presence of somatic diagnoses	Questionnaire	Self-rated	Baseline
Prevalence of potentially traumatic experiences	Life Events Checklist for the DSM-5 self-report (LEC-5) [48]	Self-rated	Baseline
Use of psychotropic drugs	Medical records	N/A	Baseline
Presence of alcohol and substance use, including prescription narcotic drugs	Biomarkers	N/A	Baseline, 6-, 12-, and 24-month follow-ups
Diagnosis of alcohol- and substance use according to ICD-10 and DSM-5 criteria	Rating form	Clinician-rated	Baseline
Severity of psychiatric illness	Clinical Global Impressions – Severity Index (CGI-S) [49]	Clinician-rated	Baseline
Depressive symptoms	Patient Health Questionnaire (PHQ-9) [50]	Self-rated	Baseline, 6-, 12-, and 24-month follow-ups
Anxiety symptoms	Generalized Anxiety Disorder − 7 (GAD-7) [51]	Self-rated	Baseline, 6-, 12-, and 24-month follow-ups
Hazardous alcohol and substance use	Alcohol Use Disorders Identification Test (AUDIT) [52, 53] Drug Use Disorders Identification Test (DUDIT) [54]	Self-rated	Baseline
Symptoms of ADHD	Adult ADHD Self-Rating Scale - Screening (ASRS-S) [55]	Self-rated	Baseline
Symptoms of insomnia	Insomnia Severity Index (ISI) [56]	Self-rated	Baseline, 6-, 12-, and 24-month follow-ups
Pain intensity and interference	Numeric rating scale for pain (NRS) [57] PROMIS Pain Interference - Short Form 4a (PROMIS-PI SF 4a) [58–60]	Self-rated	Baseline, 6-, 12-, and 24-month follow-ups
Self-rated health	EuroQol-5D (EQ-5D) [61]	Self-rated	Baseline, 6-, 12-, and 24-month follow-ups
Withdrawal symptoms	Benzodiazepine Withdrawal Symptom Questionnaire (BWSQ)[62] Subjective Opiate Withdrawal Scale (SOWS) [63]	Self-rated	Baseline
Motivation to treatment	Questionnaire (items on importance to change and self-efficacy to change) [64]	Self-rated	Baseline
Substance use last 30 days	Addiction Severity Index (ASI) [65]	Self-rated	6-, 12-, and 24-month follow-ups
Patient satisfaction with treatment	Questionnaire (items from the Swedish National Patient Survey)	Self-rated	6-, 12-, and 24-month follow-ups
Treatment at the clinic (e.g., number of visits, tapering treatment, additional treatment to tapering, diagnoses)	Medical records	N/A	6-, 12-, and 24-month follow-ups
Prescription narcotic drug use and dose	Medical records The National Prescribed Drug Register	N/A	Baseline, 6-, 12-, and 24-month follow-ups

### Statistical analysis

In the present paper, baseline characteristics and data on substance use of the final cohort of 405 participants are presented by total numbers with percentages, means, and standard deviations. The proportions presented for each variable are based on the total number of participants with complete data on the assessment or question on the instrument.

Future analyses of clinical trajectories and treatment outcomes of the cohort will be conducted using descriptive statistics and regression analysis. An analysis on the primary and secondary outcomes, including change in prescription narcotic drug use and absence of drug on supervised toxicology testing, will be presented. In the analysis on absence of drug from toxicology tests, samples will be imputed as drug positive for participants who did not attend the follow-up visit (i.e., missing data).

An a priori sample size calculation determined that at least 322 enrolled participants in the cohort were required to measure drug abstinence outcomes with 95% confidence and a margin of error of ± 5%. These calculations were based on an assumed proportion of abstinence of 0.30 [44, 66].

## Results

A total of 405 patients were included in the study. There were no significant differences in age (t-test, *p* = 0.34) or sex (chi-square, *p* = 0.73) between eligible and enrolled patients. Characteristics of the study population are presented in Table 2. The study population consisted of more women (57.5%) than men (42.5%), with a mean age of 49.2 years (SD = 14.0). At enrollment, most of the participants cohabitated with family or friends (62.0%), had completed tertiary education (56.2%), and were either students, employed, or retired (61.7%). Psychiatric disorders were common at baseline, with anxiety disorders (46.3%) and depression (40.4%) being most prevalent besides substance use disorders (81.1%). Most of the participants (72.9%) had at least two psychiatric disorders as assessed by the M.I.N.I, although the proportion varied based on the substance used at baseline: 68.5% among individuals using opioids, 83.5% for benzodiazepines, and 76.0% for benzodiazepine related z-drugs. More than half of the participants (53.8%) had a current prescription of an antidepressant. Previous treatment for problematic alcohol use or drug use were reported by 14.0% and 21.6%, respectively.

**Table 2:** Characteristics of the study population at baseline

Variable	Total sample *n* = 405
**Age** (***n* = 405)**	
Mean (SD)	49.2 (14.0)
Min, Max	20.0, 81.0
**Sex** (***n* = 405)**	
Men, n (%)	172 (42.5)
Women, n (%)	233 (57.5)
**Living situation** (***n* = 382)**	
Living alone, n (%)	131 (34.3)
Cohabitant with family/friends, n (%)	237 (62.0)
Another living situation, n (%)	14 (3.7)
**Highest education** (***n* = 381)**	
Not completed primary school, n (%)	3 (0.8)
Primary school, n (%)	53 (13.9)
Secondary school, n (%)	111 (29.1)
Tertiary school, n (%)	214 (56.2)
**Employment/occupation** (***n* = 381)**	
Employed/student/retired	235 (61.7)
Unemployed	29 (7.6)
Sick leave	64 (16.8)
Other	53 (13.9)
**Children under 18 years** (***n* = 380)**	
Yes, n (%)	115 (30.3)
**Presence of current psychiatric disorders (M.I.N.I)** (***n* = 255)**	
None	20 (7.8)
Depression	103 (40.4)
Bipolar disorder	15 (5.9)
Anxiety disorder^a^	118 (46.3)
Post traumatic stress disorder	42 (16.5)
Alcohol use disorder	45 (17.6)
Substance use disorder	207 (81.1)
Psychotic disorders	10 (3.9)
Eating disorder^b^	10 (3.9)
Antisocial personality disorder	26 (10.2)
**Comorbid disorders (≥ 2 disorders on M.I.N.I) (** ***n*** ** = 255)**	
Yes, n (%)	186 (72.9)
**Traumatic experiences during lifetime (LEC-5)** (***n* = 278)**	
Yes, n (%)	236 (84.9)
**Current prescription of antidepressants** (***n* = 405)**	
Yes, n (%)	218 (53.8)
**Previous treatment for problematic use of alcohol** (***n* = 371)**	
Yes, n (%)	52 (14.0)
**Previous treatment for problematic use of drugs** (***n* = 371)**	
Yes, n (%)	80 (21.6)

^a^Anxiety disorder include panic disorder, agoraphobia, social anxiety disorder, generalized anxiety disorder, and obsessive compulsive disorder

^b^Eating disorder include anorexia, bulimia, and binge eating disorder

Opioid analgesics (66.2%), benzodiazepines (50.9%), and benzodiazepine-related z-drugs (47.9%) were the most used prescription narcotic drugs at the time of study enrollment (see Table 3). More than half of the participants (57.5%) used at least two classes of prescription narcotic drugs: use of opioid analgesics in combination with at least one other class was the most common. The length of time study participants had used prescription narcotics varied, with a mean duration of 11.3 years (SD = 9.7). Individuals using benzodiazepines had a mean duration of use of 13.0 years (SD = 11.3) whereas individuals using opioids had use for 10.5 years (SD = 9.0), and benzodiazepine related z-drugs for 12.3 years (SD = 9.2). Most participants received narcotic drugs via prescription (75.9%), whereas almost a quarter (24.1%) received their narcotic drugs partly or completely from sources other than their health care provider. Opioid analgesics (57.8%) and benzodiazepines (43.5%) were the most common substances detected by urine and saliva, whereas use of illicit narcotics was less common in the study population (6.5%). One in ten (10.6%) had elevated levels of phosphatidylethanol (PEth), indicating harmful alcohol use. Clinician-assessed problematic use of opioids, assessed by ICD-10 and DSM-5 criteria, was the most common form of problematic use in the cohort (51.5%), followed by sedative-, hypnotic-, or anxiolytic drugs (e.g., benzodiazepines; 48.2%), tobacco (39.5%), and alcohol (12.6%). A total of 51 participants (14.9%) did not fulfill diagnostic criteria for problematic use of either opioids or sedative, hypnotic, or anxiolytic drugs. The concordance of diagnoses by ICD-10 and DSM-5 criteria, measured as percent agreement, ranged from 96.5% for sedative, hypnotic, or anxiolytic drugs to 100% for hallucinogens.

**Table 3:** Substance use at baseline, including use of prescription narcotic drugs

Variable	Total sample *n*=405
**Current prescription narcotic drug use**^a^ (***n *= 405)**	
Opioid analgesics, n (%)	268 (66.2)
Benzodiazepines, n (%)	206 (50.9)
Benzodiazepine related z-drugs, n (%)	194 (47.9)
Pregabalin, n (%)	40 (9.9)
Central nervous system stimulants, n (%)	17 (4.2)
**Current use ≥2 narcotic drug classes (** ***n *** **= 405)**	
Yes, n (%)	233 (57.5)
**Current use ≥2 narcotic drug classes including opioids (** ***n *** **= 405)**	
Yes, n (%)	185 (45.7)
**Duration of prescription narcotic drug use** (***n *= 364)**	
Mean (SD)	11.26 (9.65)
Min, Max	0.0-52.8
**Source of prescription narcotic drug** (***n *= 381)**	
Prescription from health care, n (%)	289 (75.9)
Non-prescription, other sources, n (%)	64 (16.8)
Both prescription and non-prescription, n (%)	28 (7.3)
**Positive toxicology test**,** opioid analgesics (*n* = 370)**	
Yes, n (%)	214 (57.8)
**Positive toxicology test**,** benzodiazepines (*n* = 402)**	
Yes, n (%)	175 (43.5)
**Positive toxicology test**,** illicit narcotics** (***n *= 401)**	
Yes, n (%)	26 (6.5)
**Alcohol biomarker (PEth)** (***n *= 395)**	
Harmful drinking, PEth >0.3 µmol/L, n (%)	42 (10.6)
**Current cigarette use** (***n *= 372)**	
Yes, n (%)	117 (31.5)
**Current snuff use** (***n *= 377)**	
Yes, n (%)	132 (35)
**Presence of problematic use according to diagnostic criteria** ^**b**^ **(** ***n *** **= 342)**	
None	35 (10.2)
Opioids	
Yes, n (%)	176 (51.5)
Sedative-, hypnotic-, or anxiolytics (e.g., benzodiazepines)	
Yes, n (%)	165 (48.2)
Alcohol	
Yes, n (%)	43 (12.6)
Cannabis	
Yes, n (%)	11 (3.2)
Stimulants	
Yes, n (%)	7 (2.0)
Tobacco	
Yes, n (%)	135 (39.5)
Hallucinogens	
Yes, n (%)	1 (0.3)
Inhalants	
Yes, n (%)	1 (0.3)
Opioid or Sedative-, hypnotic-, or anxiolytics	
Yes, n (%)	291 (85.1)

^a^ Current prescription narcotic drug use was based on a combination of self-report and medical records. Opioids, benzodiazepines, z-drugs, pregabalin, and central nervous system stimulants are classified as narcotic drugs in Sweden

^b^ Problematic use was based on assessment by the treating psychiatrist, according to DSM-5 and ICD-10 diagnostic criteria

## Discussion

This prospective, naturalistic cohort will evaluate tapering outcomes among individuals seeking treatment for problematic use of prescription narcotic drugs. The comprehensive data collection, which includes assessment of changes in symptoms (e.g., mood, anxiety, pain) over the course of treatment, can provide new knowledge on clinical trajectories during tapering and insight on predictors of abstinence and other treatment outcomes. The planned long-term follow-up, including a 24-month follow-up visit and a 10-year register follow-up, provide opportunities to evaluate outcomes long after completing tapering treatment. The use of objective measures of drug abstinence is a strength of this study, since self-reported abstinence may not be a reliable measure in individuals with problematic use of drugs [67, 68].

The population characteristics at baseline demonstrate that patients seeking treatment for problematic use of prescription narcotics have diverse sociodemographic backgrounds. Differences in baseline characteristics of the cohort, including narcotic drug class, and additional predictors of treatment outcomes will be further investigated in upcoming papers.

The majority of participants received their narcotics via prescription from their health care provider, with a mean use of 11 years. Opioid analgesics, followed by benzodiazepines and benzodiazepine-related z-drugs were the most frequently used prescription narcotics at baseline. More than half of the participants used at least two classes of narcotic drugs, with opioid analgesics in combination with another class being the most common. As clinical guidelines advise against using more than one central nervous system depressant due to the risk of respiratory depression and overdose death [2], it is important to decrease the proportion of patients with such potentially harmful combination therapy. This cohort will provide the ability to assess treatment outcomes in patients with polydrug use.

The baseline characteristics show a high burden of psychiatric comorbidity among individuals seeking treatment for problematic use of prescription narcotics, as recognized by clinical guidelines [2, 13], suggesting that psychological or pharmacological interventions may improve outcomes following tapering treatment. As use of prescription narcotic drugs may be a way to cope with symptoms of mental illness [69], providing alternative coping strategies might also be beneficial to increasing rates of abstinence long-term. The planned long-term follow-up in this study and information gathered on additional treatments will allow an exploratory evaluation of the effectiveness of additional interventions on outcomes.

Clinicians diagnosed problematic use in the study by assessing diagnostic criteria for harmful use and dependence (ICD-10) and substance use disorder (DSM-5), and concordance between the two sets of criteria was high. A total of 51 participants (14.9%) did not fulfill the diagnostic criteria for problematic use of opioids or sedatives, hypnotics, or anxiolytics (e.g., benzodiazepines) suggesting that patients with subclinical presentation of a narcotic use disorder may still deem their own use of prescription narcotics as problematic (e.g., had a difficult time stopping on their own), or had a referring physician who determined they required help to quit. It is important to assess patients with formal diagnostic criteria to better understand the association between illness severity and treatment outcomes.

This study has several important limitations. Although efforts were made to recruit all eligible patients that were referred to the outpatient clinic to ensure that the study population was similar to the true treatment-seeking population, slightly over one third of eligible patients declined participation or withdrew from treatment prior to giving informed consent. Although there were no significant differences in age or sex between enrolled and eligible patients in the study, there may be non-assessed differences, for example in illness severity. The exclusion of non-Swedish speaking participants and individuals with cognitive difficulties may influence the external validity of the results, however, this was a small subset of the population (*n* = 50).

Variables measured at baseline had less than 10% missing data, with the exception of current psychiatric disorders using M.I.N.I. (37%), life-time traumatic experiences (31%), and clinician-assessed problematic use (16%). Incomplete data on these assessments can affect the analysis of prognostic factors in forthcoming analyses of the cohort. The risk of missing data is in part mitigated by the inclusion of multiple assessments of psychiatric symptoms and disorders at baseline.

Supervised toxicology tests used in regular treatment were used whenever possible in order to simplify study procedures for staff and minimize the risk of data errors. Although toxicology tests to detect benzodiazepine-related z-drugs and pregabalin are measured in the study at follow-up visits, these tests were not included in the clinical routine screening at baseline, which limits our ability to characterize use of these drugs at study start. However, data on the use of benzodiazepine-related z-drugs and pregabalin was collected from self-report and medical records at enrollment.

The prospective design, large cohort size, and assessments of multiple outcomes of tapering treatment will provide novel information on prognostic factors for long-term abstinence. The study results will help provide new knowledge across several domains, including patient characteristics and treatment trajectories, that may inform future guidelines to optimize treatment for problematic use of prescription narcotics.

## Data Availability

The datasets generated and/or analyzed during the current study are not publicly available due to reasons of sensitivity but are available from the corresponding author on reasonable request.

## Abbreviations

ASI: Addiction Severity Index
ASRS-S: Adult ADHD Self-Rating Scale - Screening
AUDIT: Alcohol Use Disorders Identification Test
BWSQ: Benzodiazepine Withdrawal Symptom Questionnaire
CGI-S: Clinical Global Impressions – Severity Index
DSM-5: Diagnostic and Statistical Manual of Mental Disorders, fifth edition
DUDIT: Drug Use Disorders Identification Test
EQ-5D: EuroQol-5D
GAD-7: Generalized Anxiety Disorder − 7
ICD-10: International Statistical Classification of Diseases and Related Health Problems, 10th Revision
ISI: Insomnia Severity Index
LEC-5: Life Events Checklist for DSM-5 self-report
M.I.N.I: Mini International Neuropsychiatric Interview
NRS: Numeric Rating Scale
PEth: Phosphatidylethanol
PHQ-9: Patient Health Questionnaire
PROMIS-PI SF 4a: PROMIS Pain Interference - Short Form 4a
SOWS: Subjective Opiate Withdrawal Scale
